# Validity of the 32-item Hypomania Checklist (HCL-32) in a clinical sample with mood disorders in China

**DOI:** 10.1186/1471-244X-11-84

**Published:** 2011-05-15

**Authors:** Hai-chen Yang, Cheng-mei Yuan, Tie-bang Liu, Ling-jiang Li, Hong-jun Peng, Chun-ping Liao, Han Rong, Yi-ru Fang, Jules Angst

**Affiliations:** 1 Mental Health Institute, the 2nd Xiangya Hospital, Central South University, No. 139 Renmin Zhong Road, Changsha 410011, China; 2 Division of Mood Disorders, Shenzhen Mental Health Centre, Shenzhen 518020, China; 3 Division of Mood Disorders, Shanghai Mental Health Centre, Shanghai Jiaotong University School of Medicine, Shanghai 200030, China; 4 Zurich University Psychiatric Hospital, Switzerland

## Abstract

**Background:**

The 32-item Hypomania Checklist (HCL-32), a questionnaire for screening bipolar disorders, has been utilised in several countries, but it unclear if the Chinese version of the HCL-32 is valid.

**Methods:**

Consecutive patients with bipolar disorders (BP, *N *= 300) and unipolar major depression (UP, *N *= 156) completed the Chinese version of the HCL-32. The subjects underwent a structured clinical interview for DSM-IV Axis-I disorders (SCID).

**Results:**

The eigenvalues for the first three factors in the HCL-32 were calculated as 5.16 (active/elated), 2.72 (risk-taking) and 2.48 (irritable) using factor analysis. Cronbach's alpha for the HCL-32 was calculated to be 0.88. Positive responses to twenty-eight items were significantly more frequent by patients with BP than those with UP, and the other four items (7th, 21st, 25th and 32nd) showed no such trend. Fourteen was the optimal cut-off for discriminating between BP and UP. The HCL-32 distinguished between BP-II and UP, with 13 being the optimal cut-off. A cut-off of 13 yielded a sensitivity of 0.77 and a specificity of 0.62 between BP and UP.

**Conclusions:**

This study demonstrated that the simplified Chinese version of HCL-32 was valid for patients with mood disorders. The optimal cut-off of 13 for distinguishing between BP-II and UP was valid and could be used to improve the sensitivity of screening BP-II patients when the HCL-32 is used in psychiatric settings in China.

## Background

It is important to differentiate bipolar disorders (BP) from other mood disorders; delayed diagnosis or misdiagnosis can prolong the suffering of patients [1-3] but accurate early diagnosis can be difficult [3,4]. As many as 40% of patients with bipolar disorders are initially misdiagnosed, and it can take as long as 10 years before these patients are diagnosed correctly [4]. In the general population, the misdiagnosis rate can be as high as 69% [5]. In China, 45.4% of outpatients with bipolar disorders are diagnosed incorrectly [6]. Bipolar patients often present in the depressive phase [7] and many patients with BP (particularly bipolar II) are diagnosed as having unipolar depressive disorder [3-8]. Clinical guidelines published by the American Psychiatric Association indicate that bipolar II disorder (BP-II) is often initially misdiagnosed as a major depressive disorder, leading to patients receiving incorrect treatments [9]. Hypomania, an element of bipolar II disorder, is not usually perceived by patients to be pathological and is not reported to clinicians [10,11]. The retrospective detection of hypomania is crucial for a correct diagnosis of bipolar disorder, particularly for BP-II. An instrument to detect hypomania retrospectively would be useful in clinical settings.

Recent studies have demonstrated that the 32-item Hypomania Checklist (HCL-32) developed by Jules Angst is a good screening instrument for past hypomanic episodes [12-15]. The HCL-32 is a self-administered questionnaire that screens for a history of hypomanic symptoms using thirty-two yes/no items and takes into account the subject's current mental state. The HCL-32 was demonstrated to have good sensitivity (0.80) and specificity (0.51) at an optimal cut-off of 14, in a sample comprising predominantly outpatients with BP and UP in Europe [12]. The HCL-32 can distinguish between BP and UP at a cut-off of 14 (sensitivity 0.82 and specificity 0.67) in Taiwan [14]. However, little is known about the usefulness of HCL-32 for patients with mood disorders in China. In China, simplified Chinese characters are used, whereas in Taiwan complicated Chinese characters are used. Furthermore, in Taiwan different terms are used to express anxiety and emotion in patients. Therefore, the Taiwanese version of the HCL-32 is difficult to use in mainland China.

The aim of this study was to evaluate the feasibility of using a simplified Chinese version of the HCL-32, to examine its psychometric properties and accuracy as a screening tool for bipolar disorders. The results were compared with those from previous studies concerning the use of the HCL-32 in various countries.

## Methods

### Subjects

Subjects from the outpatient and inpatient departments at Shenzhen and Shanghai mental health centres were enrolled in the study from January 2006 to December 2008. The Shenzhen Mental Health Centre is the only psychiatric hospital in Shenzhen city. The study was approved by the ethics committees of the two psychiatric hospitals.

Patients who satisfied the inclusion and exclusion criteria were evaluated. The inclusion criteria comprised patients diagnosed with major depressive disorder (unipolar depressive disorder, UP), bipolar I disorder (BP-I) or bipolar II disorder (BP-II), aged between 18 and 60 years, educated for a minimum of five years, and who provided written informed consent. The exclusion criteria comprised patients diagnosed with an unstable or severe clinical status, those who could not cooperate with the study procedures, patients who had received electroconvulsive therapy (ECT) or modified electroconvulsive therapy (MECT) during the previous four weeks, individuals who were illiterate, suffering from mental retardation, dementia or intellectual impairment. Subjects did not have to have a certain clinical status as the aim was to elucidate the relationship between current state and HCL-32 scores.

### Measure

Upon consent from the author of the original HCL-32 (Jules Angst), the English version of the HCL-32 was translated into a simplified Chinese version. Back translation was performed by a bilingual psychiatrist unaware of the original HCL-32. A preliminary translated version was administered to individuals without psychiatric illness and patients with mood disorders. The authors reviewed the results of this preliminary investigation before producing the final version.

The contents of the HCL-32 were explained to the subjects and it was completed before the Structured Clinical Interview for DSM-IV Axis-I Disorders (SCID) was carried out; interviewers were blind to the HCL-32 results. All interviewers were psychiatrists with a minimum of five years experience. The kappa coefficient for diagnosis of bipolar disorders was 0.83.

There were contents concerning rating of current mental states (much worse than usual, worse than usual, a little worse than usual, neither better nor worse than usual, a little better than usual, better than usual, much better than usual) in the HCL-32 in addition to the 32 items [12]. Subjects were asked to select one certain state.

### Statistical Analyses

Principal component analysis with varimax rotation was used to determine the construct validity of the HCL-32. Eigenvalues > 1 were initially retained and clinical considerations decided the final number of factors. The internal consistency of the HCL-32 was determined using Cronbach's alpha. Spearman correlation analysis was performed on the current mental state and the total score. Current mental states and the mean total HCL-32 scores were compared between groups using the Kruskal-Wallis test. The frequency of each symptom item and the total HCL-32 score were compared between groups using a t-test. The receiver operating characteristic (ROC) curve was used to distinguish between groups and to ascertain the sensitivity and specificity at various cut-offs. ROC curves can be difficult to understand. Therefore, the change in sensitivity and specificity at various cut-offs are presented in figures, rather than the ROC curve. Positive predictive value was defined as the proportion of subjects screened as positive for BP using the HCL-32 and having DSM-IV BP. Negative predictive value was defined as the proportion of subjects screened as negative for BP using the HCL-32 who had DSM-IV UP. Probability values less than 0.05 were considered statistically significant. All statistical analyses were carried out using SPSS-15.0 for Windows (SPSS, Chicago, IL, USA).

## Results

### Description of samples

Four hundred and fifty six subjects (232 from Shenzhen and 224 from Shanghai), including 197 outpatients and 269 inpatients, were enrolled in the study (Table 1). The mean age of BP patients was significantly lower than that of UP patients (*t *= 5.24, *P *< 0.01).

**Table 1:** Description of samples

	UP	BP	BP-I	BP-II
N	156	300	224	76
% Female	64.10	47.33	50.45	38.16
Age (mean ± SD)	40.34 ± 14.23	33.76 ± 11.69	33.78 ± 10.67	33.15 ± 14.04
Education in years	10.21 ± 2.78	11.61 ± 3.40	11.23 ± 3.45	12.48 ± 3.11
Married, %	71.15	65.33	62.50	72.37

### Frequency of positive responses

**Figure 1:**
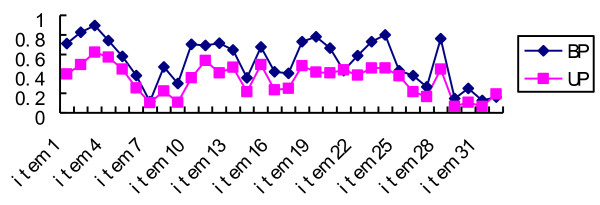
**Frequency of positive responses between BP and UP patients**. In BP patients, the frequency of positive responses to the thirty two items ranged from 11.6% (7th item, tend to drive faster) to 89.7% (3rd item, more self-confident). In UP patients, the frequency ranged from 6.4% (29th item, drink more coffee; 31th item, drink more alcohol) to 62.2% (3rd item).

The frequency of positive responses to twenty-eight items in BP patients was significantly higher than in UP patients, with the exception of four items (7th item, tend to drive faster; 21st item, more easily distracted; 25th item, more impatient/irritable; 32nd item, take more drugs; Figure 1).

### Current mental state and HCL-32 self-assessment

Mean HCL-32 scores were statistically different between groups, defined according to the current mental state of BP and UP (Table 2).

**Table 2:** HCL-32 scores (mean ± SD) for different levels of current mood state

Current mental state	BP patients		UP patients	
	
	***N***	HCL-32 score	***N***	HCL-32 score
Much worse than usual	23	13.78 ± 6.20	18	10.56 ± 6.49
Worse than usual	35	16.41 ± 5.62	31	8.97 ± 7.60
A little worse than usual	38	16.78 ± 4.99	28	10.70 ± 5.03
Neither better nor worse than usual	81	15.16 ± 7.12	46	10.35 ± 6.32
A little better than usual	37	18.19 ± 6.11	19	14.68 ± 6.28
Better than usual	44	16.59 ± 4.91	5	11.00 ± 2.65
Much better than usual	42	18.67 ± 6.45	9	15.56 ± 3.94
Significance (Kruskal-Wallis test)	-	0.04	-	0.01

A significant (*P *= 0.02) but low positive correlation (*r *= 0.13) was demonstrated between current mental state and the HCL-32 score in BP patients (*N *= 300) using Spearman correlation analysis. Similar results were obtained for UP patients (*r *= 0.23, *P *< 0.01, *N *= 156).

### Factor analysis

Analysis of data concerning subjects with mood disorders (*N *= 456) using principal component analysis with varimax rotation, revealed that the eigenvalues of seven factors were greater than 1, and this explained 51.04% of the total variance. The eigenvalues of factors I, II, and III were 5.16, 2.72, and 2.48, respectively (other factors had eigenvalues < 2). The first three factors together explained 38.34% of the total variance (Table 3). If all items suppressed absolute factor loading less than 0.35, factor I comprised 13 items (2nd, 3rd, 5th, 10th, 11th, 12th, 13th, 15th, 18th, 19th, 20th, 24th and 28th item), factor II comprised 7 items (7th, 8th, 9th, 17th, 23rd, 30th and 31st item), and factor III comprised four items (21st, 25th, 26th and 27th item). Factor I could be described as "active/elated", factor II as "risk-taking" and factor III as "irritable". Other factors for which the eigenvalues were greater than one comprised few items and were difficult to describe for each factor.

**Table 3:** Factor loadings of the HCL-32 using factor analysis (*N *= 456)

HCL-32 items	Active/elated factor loadings	Risk-taking factor loadings	Irritable factor loadings
1. need less sleep	0.32	0.17	0.2
2. more energetic	0.65*	-0.02	-0.04
3. more self-confident	0.61*	-0.08	-0.08
4. enjoy my work more	0.30	-0.11	0.02
5. more sociable	0.37*	0.03	-0.04
6. want to travel more	0.06	0.16	0.05
7. drive faster	0.06	0.50*	0.02
8. spend more	0.17	0.63*	0.07
9. take more risks	0.09	0.59*	0.10
10. physically more active	0.49*	0.08	-0.09
11. plan more activities	0.64*	-0.04	0.02
12. have more ideas/creative	0.64*	0.28	-0.04
13. less shy	0.47*	0.36*	-0.02
14. wear more extravagant clothes/make-up	0.27	0.29	0.13
15. meet more people	0.37*	0.10	0.09
16. more interested in sex	0.16	0.31	0.11
17. more flirtatious	0.19	0.36*	0.10
18. talk more	0.62*	0.12	0.27
19. think faster	0.79*	0.13	0.05
20. make more jokes	0.54*	0.26	0.04
21. more easily distracted	-0.16	0.39*	0.53*
22. engage in more new things	0.24	0.31	-0.06
23. thoughts jump	0.36*	0.52*	0.29
24. do more quickly/easily	0.66*	0.18	-0.11
25. more impatient/irritable	-0.01	0.05	0.83*
26. can be exhausting or irritating	-0.03	0.09	0.80*
27. get into more quarrels	0.07	0.24	0.64*
28. mood higher, more optimistic	0.67*	0.16	-0.07
29. drink more coffee	0.03	0.12	0.08
30. smoke more cigarettes	0.02	0.43*	0.14
31. drink more alcohol	0.06	0.37*	0.12
32. take more drugs	-0.21	0.17	0.32
Eigenvalue	5.16	2.72	2.48
Total variance explained	18.12	8.50	7.75

*:loading ≥ 0.35

### Internal consistency

Internal consistency (Cronbach's alpha) of the Chinese version of the HCL-32 was 0.88 in patients with mood disorders (*N *= 456). Cronbach's alpha of factor I, factor II and factor III were 0.88, 0.68 and 0.74, respectively.

### HCL-32 score comparison between groups

Mean HCL-32 scores of patients suffering with BP, BP-I or BP-II were statistically higher than those suffering with UP. There was no significant difference in the mean HCL-32 scores of BP-I and BP-II patients (Table 4).

**Table 4:** HCL-32 score comparison between groups

Groups	Mean HCL-32 score	***t***	***P***
BP vs. UP	16.51 ± 6.22 vs. 10.90 ± 6.43	9.05	*P *< 0.01
BP-I vs. UP	16.91 ± 6.35 vs. 10.90 ± 6.43	8.98	*P *< 0.01
BP-I vs. BP-II	16.91 ± 6.35 vs. 15.15 ± 5.92	1.88	*P *> 0.05
BP-II vs. UP	15.15 ± 5.92 vs. 10.90 ± 6.43	4.82	*P *< 0.01

### ROC curve analysis

#### ROC curve analysis between BP and UP

**Figure 2:**
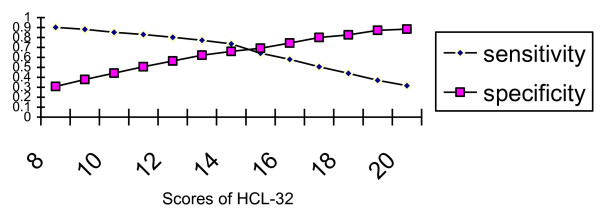
**Sensitivity and specificity at various cut-offs between BP and UP**.

ROC curve analysis revealed that the HCL-32 could differentiate between BP and UP (*P *< 0.01), and the area under the curve was 0.73. A screening score of fourteen was the optimal cut-off (sensitivity 0.74, specificity 0.66) between BP and UP. A score of thirteen yielded a sensitivity of 0.77 and a specificity of 0.62. The sensitivity and specificity at various cut-offs between BP and UP are demonstrated in Figure 2.

#### ROC curve analysis between BP-I and UP

**Figure 3:**
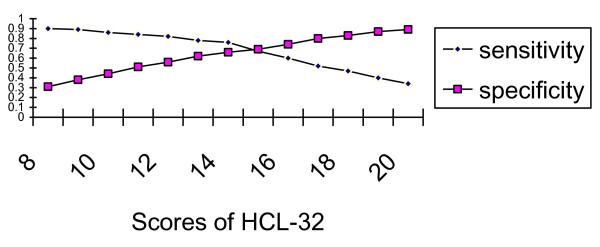
**Sensitivity and specificity at various cut-offs between BP-I and UP**.

ROC curve analysis demonstrated that the HCL-32 could differentiate between BP-I and UP (*P *< 0.01), and the area under the curve was 0.74. Fourteen was the optimal cut-off between BP-I and UP. The sensitivity and specificity at various cut-offs between BP-I and UP are presented in Figure 3.

#### ROC curve analysis between BP-I and BP-II

The HCL-32 could not distinguish between BP-I and BP-II (*P *= 0.08) using ROC curve analysis. The area under the curve was 0.57.

#### ROC curve analysis between BP-II and UP

**Figure 4:**
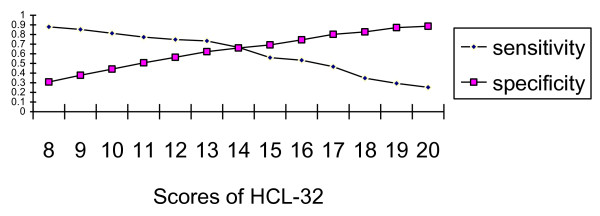
**Sensitivity and specificity at various cut-offs between BP-II and UP**. A cut-off of thirteen had sensitivity of 0.73 and a specificity of 0.62 between BP-II and UP. A cut-off of fourteen had a sensitivity 0.67 and a specificity 0.66 between BP-II and UP.

ROC curve analysis revealed that the HCL-32 could discriminate between BP-II and UP (*P *< 0.01), and the area under the curve was 0.69. Thirteen was the optimal cut-off to discriminate between BP-II and UP. The sensitivity and specificity at various cut-offs between BP-II and UP are presented in Figure 4.

### Positive Predictive Value (PPV) and Negative Predictive Value (NPV)

At a cut-off of thirteen between BP and UP, the PPV was 77% and the NPV was 56%. At a cut-off of fourteen between BP and UP, the PPV was 78% and NPV was 54%.

## Discussion

Bipolar disorder is very common and the lifetime prevalence of bipolar disorder spectrum is approximately 4.5% in the general population [16,17]. Moreover, bipolar disorder is associated with substantial impairments in productive and social roles [18,19]. The HCL-32 is a convenient instrument for screening bipolar disorders, and psychiatrists in several countries use it in practice [12-15,20,21]. China is the most populated country in the world. Therefore, a study concerning the use of the HCL-32 in China is important.

The mean age of BP patients was significantly lower than that of UP patients in this study, and this is comparable with samples used for similar studies [12,14,20]. The percentage of female UP patients was higher than the percentage of female BP patients. This could reflect the fact that rates of major depression are higher in females than in males, and they are comparable for bipolar disorder [22]. Differences concerning the mean age and sex ratio between BP and UP patients could have resulted from enrolling individuals consecutively. There were more BP-I patients than BP-II patients as inpatients as well as outpatients were enrolled in the study (more inpatients suffer from BP-I than BP-II).

The mean HCL-32 scores were statistically different between groups, defined according to their current mental state in BP and UP. Therefore, there was a possible impact of current mental state on HCL-32 scores of patients with mood disorders. This result is similar to that of a Taiwanese study [14], but different from results obtained in Europe [12,15]. Low correlation coefficients were evident between current mental state and the HCL-32 score in BP (*r *= 0.13) and UP (*r *= 0.23) patients. The impact of current mental state on the HCL-32 score is likely to be low and limited.

A three-factor solution using factor analysis in this study is different from the results obtained in the European and Taiwanese studies [12,14]. Angst reported two factors ("active/elated" and "risk-taking/irritable") from the study carried out in Europe [12]. Item 9 (take more risks) is included in factor II in the European study, but not in factor I or factor II in the Taiwanese study [14]. Combining the factor II and factor III items in the present study is similar to those of factor II in the European study. The items of factor II in the Taiwanese study are similar to those of factor III in this study [14].

Cronbach's alpha for the HCL-32 was 0.88 in the present study. This is comparable to the results from other studies (0.82 in Italian sample, 0.86 in Swedish sample, 0.90 in Spanish sample and 0.88 in Taiwanese sample) [12-15]. The internal consistency of the HCL-32 was good for various ethnic samples.

The frequency of positive responses to four items (7th, drive faster; 21st, more easily distracted; 25th, more impatient/irritable; 32nd, take more drugs) in BP patients was not significantly higher than for UP sufferers. The percentage of people who own a car in China is low, and this could explain why the frequency of the 7th item (drive faster) was low in BP (11.6%) and UP (10.3%) patients. The reason for no significant difference for the three other items is unclear.

The HCL-32 could distinguish between BP and UP, BP-I and UP, BP-II and UP, but not between BP-I and BP-II in the present study. These results are comparable to those of the European study [12]. However, HCL-32 can distinguish between BP-I and BP-II, with the optimal cut-off of 21, in the Taiwan study [14]. Subjects in the present study and that carried out in Taiwan were Chinese. In the European and Taiwanese studies, the duration criterion for hypomania was two days but in the present study it was a minimum of four days. The ratio of BP-I and BP-II patients between the Taiwanese study and the European study are similar (66/94 vs. 105/164).

In this study, fourteen was chosen as the optimal cut-off between BP and UP if BP was not divided into BP-I and BP-II. This was similar to the results from other studies [12,14]. In this study, the HCL-32 could discriminate between BP-I and UP, with the best cut-off being fourteen. In a UK study, the HCL-32 could distinguish between BP-I and UP, with the best cut-off being twenty [21].

The HCL-32 could discriminate between BP-II and UP, with the optimal cut-off of thirteen. The difficulty in distinguishing between BP and UP is related to difficulties in discriminating between BP-II and UP in psychiatric settings. Patients with BP-I are less likely to be misdiagnosed than those with BP-II. The results from the current study suggest that the optimal cut-off between BP-II and UP should be used, particularly when considering the continuum of mood disorders. BP-II is closer to UP than BP-I [23]. The sensitivity of detecting BP-II could be improved if thirteen is used as the optimal cut-off between BP and UP. There were more BP-II patients than BP-I patients [16,17,24-26]. High sensitivity is important for a screening instrument (cut-off thirteen, sensitivity 0.77, specificity 0.62; cut-off fourteen, sensitivity 0.74, specificity 0.66). From a clinical perspective, a screening questionnaire must have good sensitivity even if that increases false positives because of lower specificity [27].

The PPV at a cut-off of thirteen was 1% lower than that at a cut-off of fourteen, while the NPV was higher than 2%. The PPV and NPV at the cut-off of thirteen were better than at a cut-off of 14 but the advantage was not great.

There were limitations in the present study. The number of BP-I patients was greater than the number of BP-II patients, and there were differences in terms of the mean age and sex ratio between BP and UP patients. The duration of the mood disorders were not evaluated in the current study as diagnoses were correlated to the duration of mood disorders.

## Conclusions

The psychometric properties of the simplified Chinese version of the HCL-32 were demonstrated to be satisfactory using a clinical sample in China. The best cut-off between BP-II and UP should be regarded as the optimal cut-off between BP and UP when using the HCL-32. Furthermore, 13 can be used as the optimal screening cut-off between BP and UP in psychiatric settings in China.

## Competing interests

The authors declare that they have no competing interests.

## Authors' contributions

Authors LL and HY designed the study and developed the protocols. LL is the tutor of HY. Authors LL, HY, TL and CY carried out literature searches and analyses. Authors LL, HY, TL, HP, CL and RH undertook the statistical analysis and prepared the first draft of the manuscript. All authors were interviewers. Authors HY and TL oversaw the research in Shenzhen. Authors CY and YF directed the research in Shanghai. All authors read and approved the final manuscript.

## Authors' information

^1 ^Mental Health Institute, the 2nd Xiangya Hospital, Central South University, No. 139 Renmin Zhong Road, Changsha 410011, PR China. ^2 ^Division of Mood Disorders, Shenzhen Mental health centre, Shenzhen 518020, PR China. ^3 ^Division of Mood Disorders, Shanghai Mental Health Centre, Shanghai Jiaotong University School of Medicine, Shanghai 200030, PR China. ^4 ^Zurich University Psychiatric Hospital, Switzerland.

## Pre-publication history

The pre-publication history for this paper can be accessed here:

http://www.biomedcentral.com/1471-244X/11/84/prepub
